# Nanoparticles: Promising Auxiliary Agents for Diagnosis and Therapy of Thyroid Cancers

**DOI:** 10.3390/cancers13164063

**Published:** 2021-08-12

**Authors:** Eleonore Fröhlich, Richard Wahl

**Affiliations:** 1 Center for Medical Research, Medical University Graz, 8036 Graz, Austria; Eleonore.froehlich@medunigraz.at; 2 Institute for Clinical Chemistry and Pathobiochemistry, Department for Diagnostic Laboratory Medicine, University Hospital Tuebingen, 72076 Tuebingen, Germany

**Keywords:** cancer treatment, differentiated thyroid cancer, anaplastic thyroid cancer, endocrine cancer, mesoporous silica nanoparticles, gold nanoparticles, thyroid imaging, nanomedicine, targeted therapy

## Abstract

**Simple Summary:**

Thyroid cancer (TC) is rare relative to cancers of many other organs (breast, prostate, lung, and colon). The majority of TCs are differentiated tumors that are relatively easy to treat and have a good prognosis. However, for anaplastic TC, a rapidly growing and aggressive tumor, treatment is suboptimal because the effective drugs cause severe adverse effects. Drug delivery by nanocarriers can improve treatment by reducing side effects. This can either be mediated through better retention in the tumor tissue due to size (passive targeting) or through the attachment of specific molecules that zero in on the cancer cells (active targeting). Nanoparticles are already used for diagnosis and imaging of TC. For unresectable anaplastic TC, nanoparticle-based treatments, less suitable for deeply located cancers, could be useful, based on low-intensity focused ultrasound and near-infrared irradiation. All potential applications of nanoparticles in TC are still in the preclinical phase.

**Abstract:**

Cancers of the endocrine system are rare. The majority are not highly malignant tumors. Thyroid cancer (TC) is the most common endocrine cancer, with differentiated papillary and follicular tumors occurring more frequently than the more aggressive poorly differentiated and anaplastic TC. Nanoparticles (NP) (mainly mesoporous silica, gold, carbon, or liposomes) have been developed to improve the detection of biomarkers and routine laboratory parameters (e.g., thyroid stimulating hormone, thyroglobulin, and calcitonin), tumor imaging, and drug delivery in TC. The majority of drug-loaded nanocarriers to be used for treatment was developed for anaplastic tumors because current treatments are suboptimal. Further, doxorubicin, sorafenib, and gemcitabine treatment can be improved by nanotherapy due to decreased adverse effects. Selective delivery of retinoic acid to TC cells might improve the re-differentiation of de-differentiated TC. The use of carbon NPs for the prevention of parathyroid damage during TC surgery does not show a clear benefit. Certain technologies less suitable for the treatment of deeply located cancers may have some potential for unresectable anaplastic carcinomas, namely those based on low-intensity focused ultrasound and near-infrared irradiation. Although some of these approaches yielded promising results in animal studies, results from clinical trials are currently lacking.

## 1. Introduction

The World Health Organization (WHO) estimated in 2014 that the incidence of cancer will increase to 22 million annually within the next two decades and cancer deaths will rise from an estimated 8.2 million in 2012 to 13 million in 2032 (https://www.iarc.who.int/wp-content/uploads/2018/07/pr224_E.pdf., accessed 18 June 2021). These estimations are in line with data of the Global Cancer Statistics 2020 for 36 Cancers in 185 Countries that reported 19.3 million new cases and almost 10 million cancer death in 2020 [1]. Each of the common cancers (female breast, lung, and colorectal) represented 10–12% of the new cases in 2020 [1]. In contrast, the incidence of cancers of the endocrine system is relatively low, with thyroid cancer (TC) as the most common endocrine cancer, accounting for 3% of new cancer cases. The European Study RARECARE reported that the incidence of carcinomas of endocrine glands ranged from 4/100,000 person-years for TC to 2/10,000,000 person-years for parathyroid carcinoma [2]. 

The high frequency of cancer in the colon, breast, skin, and prostate contrasts with the low frequency in heart and small bowel. Some organs (e.g., the uterus) frequently form benign tumors and others (e.g., adrenal gland) rarely develop primary cancer at all, but host metastases of other tumors [3]. Several theories to explain the different rates of cancers in different organs have been proposed. In addition to the number of stem cell divisions (increasing the risk of mutations), the evolutionary antiquity of an organ (longer time to optimize preservation of organ function), and the role of external factors (smoking), Thomas et al. postulated the role of the organ for survival in cancer development as a relevant parameter. According to this theory, paired, large organs that are not essential for survival can tolerate a higher amount of (generally non-functional) tumor cells than small, unpaired organs essential for living. The thyroid would fulfill the requirements of an organ with a low risk for cancer development according to all theories because it is evolutionarily old, with a medium rate of stem cell divisions, unpaired, small, and essential for survival. Chordates 600 million years ago already possessed a thyroid gland, which contrasts with the first appearance of the mammary gland in monotremes 170 million years ago and the prostate in marsupials 150 million years ago [4]. Stem cell divisions in the thyroid occur at a rate of 0.087/year compared to the large intestine with 73/year [5]. The theories fit less well for lung adenocarcinoma, where external factors (smoking) play a major role in cancer development. The role of mutation is not convincing because, in the absence of specific inducers, no correlation between the number of gene mutations and cancer risk across tissues has been reported [6]. It appears likely that the low incidence of endocrine cancers and their generally low malignancy is due to the inherent properties of the endocrine cells themselves, because the microenvironment per se does allow the growth of cancers [7]. Thus, melanoma, lung, and breast cancer metastasize to the endocrine glands, frequently the pituitary and adrenal glands. Except for the thyroid gland, metastasis causes endocrine dysfunction.

## 2. Thyroid Carcinoma

The majority of TC are papillary carcinomas (PTC, 80%), follicular carcinomas (FTC, 10%), and medullary thyroid carcinomas (MTC, 5–10%). Anaplastic carcinomas (ATC) represent 1–2% of thyroid malignancies and primary thyroid lymphomas and primary thyroid sarcomas are extremely rare. Hürthle cell carcinoma is considered a variant of follicular carcinoma [8]. Classification of the different subtypes of TC is outside the scope of this review and the reader is referred to expert reviews on this topic (e.g., [9,10,11]). The origins of the tumors are either the epithelial cells of the thyroid follicles (in PTC, FTC, and ATC) or the parafollicular cells, which produce the hormone calcitonin (in MTC). MTC belongs to the group of neuroendocrine tumors (NETs) derived from cells that have the unique ability to synthesize, store, and secrete a variety of metabolically active substances, peptides, and amines and can be located in various parts of the human body [12]. The majority of the differentiated TC, PTC, and FTC are not highly aggressive and invasive tumors, and nearly all NETs of the pancreas (insulinoma, glucagonoma, and gastrinoma) and pheochromocytoma are not malignant [13]. 

The prognosis of differentiated TC is very good and in patients younger than 50 years of age, both PTC and FTC have a more than 98% cure rate [14]. MTC have a worse prognosis because it metastasizes to lymph nodes at an early stage and requires extensive surgery. ATC is usually diagnosed after it has already spread and is one of the most incurable cancers. The tumors can be very complex, with 20–30% of ATC patients having tumor areas with ATC and PTC histology [15]. Malignant bone and cartilage formation has also been observed and the descriptive term “carcinosarcoma” as subtype of ATC has been suggested by the WHO but this classification is not uniformly accepted. One reason for that is the fact that ATC are of epithelial origin, while carcinosarcomas show cells of the epithelial and mesenchymal lineage [16]. The term “coexisting carcinoma and sarcoma” was deemed better to describe the variable progression of the two components. Progression from poorly differentiated PTC to ATC has been reported in 20% of ATC patients and, according to the American Thyroid Association, is a risk factor for ATC [17].

Treatment of differentiated TC according to the guidelines of the European Society of Medical Oncology (ESMO) includes thyroidectomy with different extents of lymph node resection (ranging from no to radical neck dissection) followed by radioiodine treatment with ^131^Iodine [18]. Radioiodine treatment can also be used as the only treatment option. Prior to the treatment, iodine uptake is stimulated either by withdrawal of levothyroxine or by stimulation with recombinant TSH [19]. Treatment of TC has been unified based on collaboration between the American Thyroid Association, the European Association of Nuclear Medicine, the Society of Nuclear Medicine and Molecular Imaging, and the European Thyroid Association.

**Figure 1:**
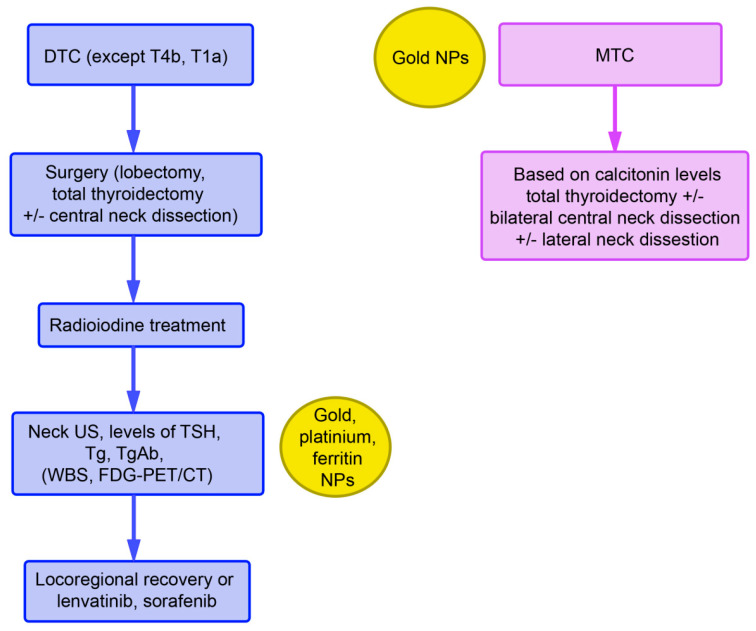
Recommendations of the ESMO for the treatment of differentiated TC (DTC) and MTC and indications for nanoparticles (NPs) that could be used at the respective diagnostic/treatment phase. Abbreviations: FDG-PET/CT, [^18^F] fluorodeoxyglucose (FDG) positron emission tomography (PET)/computed tomography (CT); Tg, thyroglobulin; TgAb, thyroglobulin antibodies; TSH, thyroid stimulating hormone; US, ultrasound; WBS, whole body scan.

Monitoring after radioiodine treatment includes thyroglobulin (Tg) and anti-Tg antibody levels, in addition to TSH levels. Tg measurement is the most sensitive parameter for persistent and/or recurrent disease. Tg antibodies can interfere with the measurement and yield evidence of functional thyroid cells. Elevated levels may indicate recurrent/metastatic disease. Neck ultrasound should also be included in routine monitoring. Whole-body scans (WBC) with a radioactive iodine tracer as well as [^18^F]fluorodeoxyglucose positron emission tomography (PET)/computed tomography (CT) can also be used. In the case of a suboptimal response, administration of lenvatinib and sorafenib is recommended (Figure 1). 

**Figure 2:**
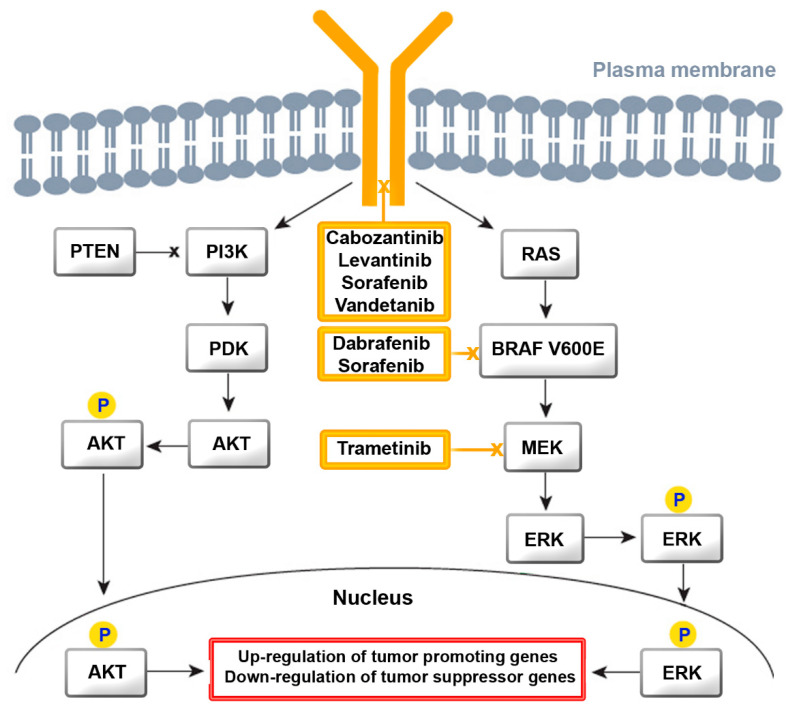
Signaling cascade of the receptors for growth factors, with the points of action for the most established tyrosine kinase inhibitors (TKIs) shown for TC. Activation of kinases by phosphorylation is indicated by “P”. Abbreviations: BRAF V600E, V-raf murine sarcoma viral oncogene homolog B; MEK, mitogen-activated protein kinase/ extracellular signal-regulated kinase; ERK, extracellular signal-regulated kinase; PTEN, phosphatase and tensin homolog deleted on chromosome 10; PI3K, phosphatidylinositol 3-kinase; PDK, 3-phosphoinositide-dependent kinase; RAS, rat sarcoma virus.

Both of these drugs are tyrosine kinase inhibitors (TKIs) for multiple receptors, which include receptor tyrosine kinases (RTK) and growth factor receptors (GFR). They inhibit kinases of the vascular endothelial growth factor receptor (VEGFR) 1–3 and of RAF, V-raf murine sarcoma viral oncogene homolog B (BRAF), platelet-derived growth factor receptor (PDGFR), cKIT, FMS-like tyrosine kinase-3 (FLT3), fibroblast growth factor receptor (FGFR) 1–4, and RET for proliferation and normal cell function. GFR signaling acts via the rat sarcoma virus (RAS)/RAF/Mitogen-activated protein kinase/extracellular signal-regulated kinase (MEK)/extracellular signal-regulated kinase (ERK) cascade (Figure 2) [20]. Binding of a ligand to RTK is the key activation step for the RAS cascade. In parallel, the ERK cascade is also activated. In turn, increased activity of RTK, RAS, and RAF activate the mitogen-activated protein kinase (MAPK) pathway, resulting in constitutive activation of MEK and ERK. Regarding the phosphatidylinositol 3-kinase (PI3K)/AKT pathway, AKT is activated by the conversion of phosphatidylinositol bisphosphate to phosphatidylinositol trisphosphate via PI3K. Phosphatase and tensin homolog deleted on chromosome 10 (PTEN) acts as a negative regulator of this pathway by catalyzing dephosphorylation. Several TKIs inhibit the downstream steps. Dabrafenib and sorafenib for mutated BRAF and tramentinib for MEK are of relevance for ATC treatment [21]. 

TKIs have been approved for treating differentiated TC, MTC, and ATC. Their use should be considered after careful weighing of the potential risks and benefits of this therapy. Lenvatinib has serious adverse effects on the cardiovascular system, liver, and renal function. Sorafenib use is associated with severe skin adverse events, hemorrhage, and cardiovascular effects. A systematic review by Fleeman et al. found that improvements in progression-free survival and objective tumor response rate were accompanied by increased risk of adverse effects [22]. The effect on overall survival and health-related quality of life remains uncertain. MTCs are treated according to calcitonin levels with total thyroidectomy, bilateral central neck dissection, and lateral neck dissection. The multi-kinase TKIs vandetanib and cabozantinib show real-world efficacy and safety in the treatment of progressive MTC and will potentially be included into the recommendations of ESMO in the future [23].

**Figure 3:**
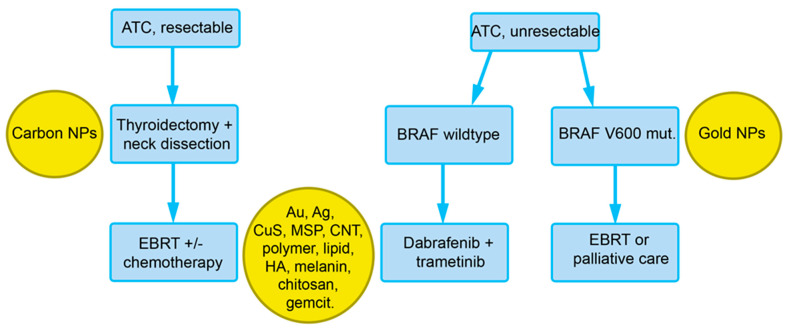
Overview of the recommendations of the European Society for Medical Oncology (ESMO) for the treatment of ATC with indications for nanoparticles that could be used at the respective diagnostic/treatment phase (see Section 4). Abbreviations: Ag, silver; Au, gold; BRAF V600E, V-raf murine sarcoma viral oncogene homolog B; CNT, carbon nanotubes; EBRT, external beam radiation therapy; HA, hyaluronic acid; gemcit, gemcitabine; MSP, mesoporous silica particles.

For poorly differentiated tumors and ATC, total thyroidectomy + neck dissection followed by external beam radiotherapy (EBRT) ± chemotherapy is performed (Figure 3). In cases of unresectable tumors and BRAF V600E mutation, dabrafenib + trametinib, and in unresectable tumors with BRAF (wildtype), EBRT or palliative care is indicated. Dabrafenib and trametinib are prescribed preferentially as a combination, also in non-small cell lung cancer and melanoma [24]. In one trial, where patients with de-differentiated TC might also have been included, a response rate of 70% has been reported [25]. These TKI may cause adverse effects, such as a lack of appetite, rash, and hypertension. Severe pyrexia upon therapy with trametinib is particularly serious and difficult to treat and may warrant discontinuation of the drug. 

The TKIs playing an established role in the treatment of TC act on RET, FGFR, cKIT, PDGFR, VEGFR (lenvantinib), BRAF, RET, c-KIT, PDGFR, VEGFR (sorafenib), BRAF (dabrafenib), MEK (trametinib), RET, VEGFR (cabozantinib), and RET, EGFR, and VEGFR (vandetanib) [26,27]. 

## 3. Nanomedicine in Cancer

The term “nanomedicine” has been coined by Dr. Robert Freitas in 1994 and was defined in 2004 by the European Science Foundation (ESF) and in 2006 by the United States’ National Institutes of Health (NIH). Both reports emphasize that nanomedicine emerged from nanotechnology, which is generally defined by the creation and use of materials at the level of molecules and atoms (sometimes specifically less than 100 nm, other times this dimension is more diffuse and confusing) [28]. Vanishingly few (4) articles were obtained from a literature search in PubMed with the key words “nanomedicine” AND “cancer” NOT “review” during the period 2000–2005, while 9600 articles with these key words have been published from 2006 up to the time of writing. The translation of engineered particles to the clinics is slow. Submissions to the Food and Drug Agency (FDA) represent liposomes (33%), nanocrystals (23%), emulsions (14%), iron-polymer complexes (9%), and micelles (6%), and the approval of nanomedicine products is 1–7/year [29]. With 35% of the submissions, cancer presents the main application area for nano-based products. NPs should improve the delivery of chemostatic drugs with poor water solubility, rapid metabolization/excretion, or show high off-target action, such as doxorubicin, paclitaxel, cisplatin, and gemcitabine [30]. They can also carry contrast agents for imaging or serve as sensors. 

Commonly used NPs in cancer are organic (liposomes, polymer-based, and dendrimers), inorganic (gold, carbon-based, mesoporous silica, magnetic, and quantum dots), and hybrid (liposome-silica, chitosan-carbon, and cell membrane-coated) particles [31]. They deliver chemotherapeutics, gene/siRNA, photosensitizers, and thermal energy for treatment. Further, NPs serve for imaging and for detection of biomarkers. Co-delivery of various anti-tumor agents or of anti-tumor agents and contrast agents is another advantage of NP delivery.

A literature search for original articles in the PubMed data base using “nano*” AND “cancer” AND “gold” or “quantum dots” or “mesoporous silica” or “iron oxide” or “carbon” or “liposomes” or “polymeric” showed that most studies used gold NPs (6200), followed by carbon-based materials (3700), polymeric (3000), liposomes (2600), and iron oxide NPs (2200). It appears not surprising that most studies focused on gold NPs because this material is suitable for drug delivery and sensor technology [32]. 

**Figure 4:**
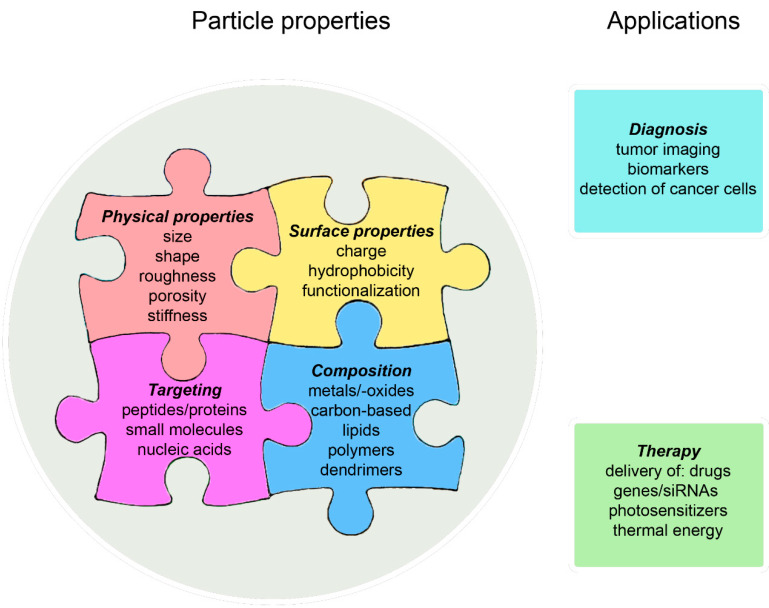
Nanoparticle properties—important for their biological effects and their potential indications in cancer.

The biological effects of NPs are determined by a panel of interacting parameters, namely, their composition, physical properties, surface properties, and targeting molecules, and it is difficult to predict the influence of variation in one particular parameter on the overall effect (Figure 4). Information on the importance of these parameters for the biological action of NPs is provided elsewhere (e.g., [33,34]). Nanotechnology in cancer aims to improve tumor-specific delivery of approved drugs using various strategies.

### 3.1. Passive Tumor Targeting

It is hypothesized that NPs have an inherent capacity to accumulate in tumors due to the enhanced penetration and retention effect (EPR). This theory was formulated in 1986 and proposes that leaky vasculature in combination with defective lymphatic drainage facilitates particle accumulation in tumors [35]. Vascular permeability, receptor expression, and vessel maturation are additional factors. The upregulation of VEGFR2 makes blood flow sluggish in the tumor. Fenestrations and decreased lymphatic drainage lead to high interstitial fluid pressure, which compresses blood vessels. This situation leads to a greater retention of the NPs in the tumor tissue. Diffusion of the particles to the tumor cells may be hindered by the increased collagen content of the extracellular matrix and by uptake by macrophages. This effect has been shown consistently in subcutaneous xenografts of mice but the effect in humans is not undisputed [36]. One reason for doubt is the difference between the theoretically expected lower extravasation of particles in the tumor periphery with more intact vasculature compared to the necrotic core with defective vasculature. This expectation contrasts with the opposite findings in vivo. The EPR effect appears to be heterogeneous in tumors, to occur only transiently and to differ between patients [37]. Part of the inter-tumor heterogeneity can be explained by variable hypoxia, interstitial fluid pressure, cellularity, and extracellular matrix density. Pre-screening of the patients prior to administration of liposomal drug formulations may be useful because liposomal deposition may range from undetectable to 53% injected dose/kg or vary by a factor of 38 [38]. Determination of the extent of the EPR effect is important because it is positively linked to the success of the nanoformulations. The use of ^64^Cu-liposomes in PET is suitable for the screening and could serve for patient selection. Drug delivery by NPs does not appear to be high in absolute terms. A meta-analysis based on 117 publications reported that on average 0.7% of the administered nanoparticle dose reached the tumor cells of solid tumors [39]. Although data from TC were not included in this study, it is reasonable to assume that delivery would not differ from the evaluated cancers (brain, breast, cervix, colon, liver, lung, ovary, prostate, and skin). The authors noted that inorganic material, smaller (<100 nm) size, neutral zeta potential, rod shape, and active targeting lead to slightly higher delivery rates. They also reported that the choice of the mouse model (orthotropic vs. xenograft) had an influence on the performance of the nanocarriers. In order to illustrate the advantage of nanocarriers, the efficacy of paclitaxel by poly(ethylene oxide)-surface modified poly(d,l-lactic-co-glycolic acid)/poly(beta-amino ester) polymer-blend NPs is used. The NPs delivered 0.6% of the injected dose, whereas only 0.2% of the free drug was detected in the tumor [40]. The EPR effect can be increased by radiotherapy, hyperthermia, and sonoporation (efficiency enhanced by microbubbles as contrast agents). By monitoring the accumulation of magnetic carboxymethyl-dextran-coated iron oxide NPs in the tumor by magnetic resonance imaging (MRI), the extent of the passive tumor targeting can be estimated [41]. 

### 3.2. Active Tumor Targeting

In addition to passive targeting by the EPR effect, active targeting by the attachment of the ligands can be used. This strategy is useful because the transformation to cancer cells is linked to changes in the expression of the surface molecules. A variety of surface molecules are overexpressed in cancer cells and the tumor microenvironment. Ligands or antibodies targeting these molecules can be attached to NPs to achieve tumor-specific delivery. Due to its greater stability, covalent conjugation is often preferred to physical absorption of the ligand to the NP [30]. Two ligands instead of one can increase affinity but ligand density is not linearly correlated to binding affinity because of interference by improper ligand orientation, bond constraints, and steric hindrance from neighboring molecules on the NP. The binding of small proteins and biomolecules in the extracellular matrix (so-called Vroman’s effect) can decrease the specificity of the targeting. A model based on diffusivity, permeability, available volume fraction, and plasma clearance predicted that active targeting is efficient for particles up to 50 nm [42]. Targeting with high molecular weight molecules is suboptimal because of the risk of enzymatic degradation of the targeting molecules and lack of ability of the coated NPs to cross membranes. Extracellular release of the payload can be achieved by redox reaction/oxidation, or can be pH-mediated, or triggered by magnetic fields, photoinduction, ultrasound, electrochemically, or by temperature change. On the cellular level, delivery to the intended compartment is important. NPs are usually taken up actively by endocytosis and transported to the lysosomes [43]. The subsequent release of the payload from the lysosomes and prevention of release into the extracellular space by exocytosis represent major challenges. Exocytosis of NPs has been determined using loading of cationic lipidoid C12-200 particles with siRNAs labelled with two different fluorochromes and exploiting the techniques of fluorescence resonance energy transfer (FRET) [44]. FRET functions only if the fluorochromes are close together in the lipid particle and the decrease in the signal indicated that this was no longer the case after 1 h. Fluorescence of the single dyes remained constant and fluorochrome was detected in the supernatant. Based on these findings, the authors concluded that the NPs disintegrated in the cells and that ~70% of the NPs were exocytosed and only 1–2% of siRNA was released from the lysosomes. The extent of exocytosis cannot be generalized because it is influenced by the particle characteristics, cell type, and medium [45].

Tumor-specific targeting requires a specific, highly, and homogeneously expressed tumor antigen. Targets for tumor-specific targeting to several tumors are the epidermal growth factor receptor (EGFR), folic acid receptor, lectins, holo-transferrin, hyaluronic acid, cell type-specific peptides encoding the arginine, glycine, and aspartate (RGD) sequence, or antibodies against CD22 or CD30. Nanoalbumin-bound paclitaxel (nab-PTX) interacts with the secreted protein acidic and rich in cysteine (SPARC) overexpressed by invasive tumors and metastases. BIND-014 is specific for prostate cancer; this is a docetaxel-loaded polymeric NP that targets the prostate-specific membrane antigen [46]. Targeting of the endothelium is achieved by using the αvβ3 integrin and vascular cell adhesion molecule-1 (VCAM-1), and of the extracellular tumor stroma by matrix metalloproteases (MMPs) [31]. Exploitation of the homing behavior of cells (e.g., T-cells) is a biological targeting mechanism [47]. More recently, the use of biological membranes, tumor cell membranes, or membranes from extracellular vesicles for delivery of chemotherapeutic and immunomodulatory agents has been established. Their properties compared to other biological NPs have been summarized in a recent review [48]. The use of biological membranes for targeting has the advantage of lower protein binding to the particle surface (protein corona), which decreases the circulation times for engineered NPs functionalized with ligands. Protein corona formation is prevented by coating the NPs with polyethylene glycol (PEG). This material may cause allergies; however, mainly upon oral administration (81%) and rarely on parenteral administration (16%), which is more common in cancer treatment [49]. The need for pegylation is not completely clear because NPs may not need long circulation times for accumulation in large tumors with high blood flow [41]. 

The use of NPs can be classified into diagnostic and therapeutic use. The studies are summarized in Table 1 and discussed in Section 4 and Section 5. 

## 4. Nanoparticles in the Diagnosis of TC

Biomarkers can help in diagnosis or for disease monitoring. Their use in monitoring of TC is more important because the search for specific biomarkers so far has not been successful. Markers related to metabolism, thyroid function, and tumor phenotype have been identified but the majority were general cancer markers [85]. BRAF V600 and calcitonin levels have a role in the treatment strategy (Figure 1) but for other hormones a lack of clear cut-off values presented the greatest problem. Recently, the first markers for ATC have been reported [86]. Melanoma-associated antigen A3 (MAGEA3) and the oncofetal IGF2 mRNA binding protein 1 (IGF2BP1) were suitable for differentiation between ATC and undifferentiated PTC and IGF2BP1 may have the potential to serve as a prognostic marker. 

### 4.1. Circulating Biomarkers

Liquid biopsies are useful tools to detect biomarkers, such as circulating tumor cells, circulating vesicles (extracellular vesicles), circulating nucleic acid, and circulating proteins derived from tumor cells. Gold NPs are the most often used NPs because they offer detection by fluorescence, colorimetry, photoacoustics, surface enhanced Raman scattering (SERS), electrochemistry, dynamic light scattering (DLS), or localized surface plasmon resonance (LSPR) [32].

Anti-TSH antibody-conjugated horseradish peroxidase (HRP) immobilized on platinum (Pt) NPs with detection by chemiluminescence was developed for the sensitive detection of TSH, useful for instance in the monitoring of TC [50]. This assay was three times faster and 100 times more sensitive than commercially available assays. Another sensor, in which polyamidoamine dendrimers enlarged the binding sites on a gold electrode, was able to detect TSH with a limit of detection of 0.026 mIU/L [51]. Third-generation TSH assays have a sensitivity of 0.1–0.02 mIU/L and fourth-generation immunochemiluminometric assays can detect TSH levels in the range of 0.01–0.001 mIU/L [87]. The latter are rarely used because the sensitivity of the third-generation assays is generally sufficient in clinical practice.

A novel fluoroimmunodiagnostic nanoplatform using tannylated ferritin nanocages was developed for Tg [52]. Fluorescein-5-isothiocyanate (FITC)-labelled anti-Tg antibodies were conjugated to nanocages via multiple hydrogen bonds and hydrophobic interactions. The lower limit of detection was 4.3 pg/mL in artificial human serum medium, which is lower than existing assays, for instance the Elecsys Roche Tg II assay and the ultrasensitive Tg with 0.04 ng/mL [88]. However, increased sensitivity is not urgently needed for these assays because, again, currently available assays already fulfill the clinical requirements. 

Sensors have been developed for detection of calcitonin as a marker and important parameter for a treatment decision for MTC [89]. Flower-like thin-film gold nanoparticles doped in a sol-gel/polyethylene glycol mold were designed with sensing based on FRET. The quenching of the fluorescence intensity by calcitonin had a detection limit of 0.707 pg/mL [53]. Gold nanoparticles and graphene oxide on the activated surface of a glassy carbon electrode improved the loading of the anti-calcitonin capture antibody and resulted in enhanced sensitivity [54]. The signal detection is based on the shift of the peak current resulting from the change in surface charge due to the antigen–antibody sandwich-type immunoreaction. The detection limit for calcitonin was 0.7 pg/mL. Both nano-based assays achieved similar limits of detection (1.0 pg/mL), but these were lower than commercial assays (https://www.quidel.com/sites/default/files/product/documents/8043_2.pdf, accessed on 10 June 2021).

BRAF mutations are frequent in PTC (~60%) and in dedifferentiated PTC. These mutations can be detected in DNA extracted from peripheral blood, plasma, serum, circulating tumor cells, and circulating free DNA. Circulating tumor cells can be enriched from the blood of patients suffering from several common cancers (lung, liver, breast, prostate, and colon) with magnetic NPs [90]. Positive and negative magnetophoretic isolation is possible with tumor cells expressing epithelial cell adhesion molecule (EpCAM) as the most widely used antigen for positive selection and anti-CD45 for the depletion of white blood cells as a negative marker. EpCAM is also used for the isolation of circulating tumor cells in metastatic PTC but the antigen is not expressed on ATC cells [91]. Ferrofluids, usually consisting of Fe_3_O_4_, are stable colloidal suspensions containing a single magnetic domain with a diameter of about 10 nm. Screening for mutations in ATC is important because ATC with wild-type BRAF are treated with TKIs, while patients carrying tumors with the BRAF V600 mutation receive EBRT or palliative treatment (Figure 3). Enrichment of circulating ATC cells is currently not possible because no specific ferrofluid exists. 

Free circulating DNA can be isolated with conventional tests. It has a size of about 40–200 bp if derived from apoptotic cells and 20–30 kb from necrotic cells. The fraction of tumor DNA in the samples varies between 0.01 and 10% [92]. Several techniques can be used for the detection of mutated BRAF and the lower limit of detection (LOD) is expressed as the number of wild-type copies per one mutated gene detected. Sequencing techniques (Sanger sequencing, pyrosequencing, and next generation sequencing) are less sensitive than matrix-assisted laser desorption ionization–time of flight (MALDI-TOF) and polymerase chain reaction (PCR)-based techniques. The most sensitive identification of mutations uses digital PCR, where assays have LODs between 0.0005 and 0.0045% [93]. BRAF V600 and KRAS exon 2 ctDNA assays have a LOD of 0.01% and 0.02%, respectively [94]. Gold NPs were used to increase the sensitivity of mutated BRAF gene detection by immunochemistry [55]. The 30-nucleotide DNA probe was immobilized in a streptavidin-modified microtiter plate and the biotinylated target DNA added. After addition of streptavidin-labeled gold NPs, a nanoparticle enlargement process was performed using a gold ion solution and formaldehyde reductant. The gold particles were then dissolved in bromide and the DNA hybridization detection process was performed using a square wave stripping voltammetry (SWSV) technique. The gold-amplified biosensor had a LOD of 0.35 amol/L [55]. Another sensor was based on S-regulated boron nitride quantum dots, which present significantly different electrochemiluminescence (ECL) properties and electro-optical activity. A ratiometric and enzyme-free ECL sensing mode was constructed with the amplified surface plasmon-coupled ECL strategy. The proposed DNA sensor has a limit of detection of 0.3 pmol/L [56]. For these genetic studies, increased sensitivity may improve diagnosis. Although the detection limits were much lower than the limits of available assays, the less common protocols and platforms represent a hindrance for fast translation into clinical practice.

Fine Needle Aspiration Biopsy is the gold standard for evaluation of suspicious thyroid lesions, although differentiation between FTC and follicular adenoma or between oncocytic thyroid adenoma and Hürthle cell carcinoma pose problems. Nanotechnology may provide an additional tool by proteomic analysis of the patient-specific protein corona on gold, silver, and iron NPs [95]. The protocol uses formalin-fixed, paraffin-embedded samples for extraction of the protein, coating of the NPs, separation of the proteins by electrophoresis, digestion of the proteins in the gel, and identification by mass spectrometry.

### 4.2. In Vivo Imaging and Combination of Imaging and Therapeutics (Theranostics)

Common technologies in diagnosis, staging, and management of TC include ultrasound, CT, and MRI [96]. The role of FDG-PET is less clear. ^131/123^I whole body scans can be used to detect residual locoregional disease in the neck and distant metastases. It is ideally combined with 3D conventional imaging using single photon emission computed tomography (SPECT) in combination with CT. [^18^F] FDOPA and [^68^Ga]DOTATOC are the preferred tracers for MTC [97]. Primary TC is located near the body surface and detection may profit from specific techniques less suitable for deeply located tumors due to the low penetration depths of fluorescent light and ultrasound [98]. Photothermal treatment, which relies on the absorption of NIR light and conversion to thermal energy, can be effective to a depth of 2–3 cm below the skin [99]. The penetration depths of ultrasound are difficult to indicate because absorption is tissue specific (e.g., low absorption in blood and fat), and the thicknesses of the tissue layers in individual patients are unknown. The maximum depths to achieve effective heating at a 2.5 MHz frequency of unfocused ultrasound have been indicated as 3–6 cm [100].

Imaging techniques have specific advantages and limitations and the combination of two strategies is generally recommended. NIR fluorescence imaging is characterized by great sensitivity but poor resolution. CT, by contrast, has a high spatial resolution but low sensitivity. By using gold NPs for imaging, both techniques can be combined. In an orthotopic xenograft mouse model using human thyroid cancer patient tissue, dual imaging for detection of TC cells was possible with iodinated gold nanoclusters synthesized via bovine serum albumin and chloramine-T [57]. Coating with BSA provided stabilization and better tissue penetration. The particles were able to detect implanted human TC as small as 2 mm^2^ in nude mice. The uptake of ^131^I-VEGF-targeted mesoporous silica nanoparticles (MSNs) by the xenografted ATC cells resulted in a strong SPECT signal after intratumoral injection [58]. If the particles were injected via the tail vein, however, they accumulated in the mononuclear phagocyte system (MPS).

Low-intensity focused ultrasound (LIFUS) can be used to combine imaging and treatment of superficially located tumors. The technology consists of sonodynamic therapy, ultrasound-mediated chemotherapy, ultrasound-mediated gene delivery, and anti-vascular ultrasound therapy [101]. There is no widely accepted definition of low-intensity focused ultrasound. One suggestion is to use insonation with an intensity less than 5.0 W/cm^2^. The commonly used microbubbles have the disadvantage that they are too big for targeting to markers. NPs, which allow the targeting, have worse acoustic responsiveness but this limitation can be overcome by inclusion of phase-transformable liquid fluorocarbon, which has the property of transforming from the liquid phase to gas under irradiation [102]. Src homology 2 domain-containing phosphotyrosine phosphatase 2 (SHP2) is a proven oncogene for TC, and phase-changeable polylactic-co-glycolid acid (PLGA) polymeric nanoparticles decorated with SHP2 antibody were able to strongly label tumor tissue when applied to tumor-bearing mice and irradiated with LIFUS [59]. LIFUS based on targeted contrast agents results in tumor-specific cell damage in contrast to highly focused ultrasound, which also damages normal tissues [103]. 

The combination of PET imaging and photothermal therapy can be performed with PEG-coated copper sulfide ([^64^Cu] CuS) NPs [60]. Because the light penetration is better, the longer the wavelength of the light, excitation of CuS NPs with 1275 nm is preferred to its excitation at 808 nm [104]. In an orthotopic nude mouse model of ATC, [^64^Cu] CuS NPs were injected into tumors derived from 5 × 10^5^ luminescent Hth83 cells (ATC cells transfected with luciferase) and the mice underwent a PET scan to localize the tumor [60]. Tumor growth was evaluated after intravenous injection into orthotopic Hth83 tumor-bearing mice with and without NIR irradiation at 930 nm for combination treatment. Although only ~6% of the injected NPs reached the tumor at 24 h, the combined treatment resulted in a tumor reduction of up to 83%. Systemic toxicity was absent. Anti-tumor activity is markedly higher upon combined radiotherapy and NIR irradiation than with either therapy alone. NPs enable the combination of imaging agents and therapeutic molecules, commonly described using a combination of the words therapeutic and diagnostic to indicate “theranostic” particles. Novel NPs were designed from poly[2,6-(4,4-bis-(2-ethylhexyl)-4H-cyclopenta [2,1-b;3,4-b′]dithiophene)-alt-4,7(2,1,3-benzothiadiazole)] (polyPCPDTBT) with both excitation and emission in the NIR region. These polymeric NPs facilitated tumor imaging. The application of BRAF silencing siRNA-loaded NPs identified xenograft tumors after injection of 2 × 10^6^ BRAF V600E-mutated 8505C cells (established from an undifferentiated human TC) in athymic nude mice or orthotopic tumors after implantation of 5 × 10^5^ BRAF V600E-mutated 8505C cells in SCID mice [61]. The polymer enables hydrophobic interaction with the amphiphilic cationic lipids that are widely used for complexing with siRNA, and a siRNA encapsulation efficiency of ∼50% can be achieved. In addition to localization of the tumor cells, the particles significantly suppressed tumor growth and metastasis in the orthotopic mouse tumor model.

The future of NPs in diagnosis is tightly linked to the availability of specific TC tumor markers. If such markers are identified, there will be the possibility to optimize their detection. The option to identify new markers by use of the patient-specific protein corona [95] may present a promising yet not very fast tool to improve diagnosis of TC.

## 5. Nanoparticles in the Treatment of TC

NP-based treatments were mainly developed for ATC rather than all TC because differentiated TC can be treated specifically with ^131^Iodine (Figure 1); the NPs for ATC treatment are listed in Figure 3. With the exception of the carbon nanoparticles used in surgery, NPs developed for ATC are still in the phase of preclinical testing (cell and animal studies).

### 5.1. Inorganic Nanoparticles: Carbon Nanoparticles

Lymph node metastasis in PTC occurs in 20–50% of cases and is an important factor in recurrence. The goal of total thyroidectomy in TC is the ablation of the thyroid and an optimal number of lymph nodes without damaging other structures in the neighborhood of the thyroid, mainly the parathyroid glands and the recurrent nerve. Transient or permanent hypothyroidism after surgery was reported to occur in up to 37% of thyroidectomies [105]. Carbon nanoparticles of diameter 150 nm enter lymphatic vessels but not blood vessels and are transported to regional lymph nodes [106]. Their intratumoral injection may help to discriminate lymph nodes from parathyroid glands during thyroid surgery and should decrease the rate of hypoparathyroidism caused by removal of the parathyroid glands or damage to the blood supply. Parathyroid glands with vasculature and recurrent laryngeal nerves are not stained and thus remain the primary color, while abnormal thyroid tissue and lymph nodes stain black. Although on average more lymph nodes were identified by using the carbon nanoparticles, there was no significant difference in the rate of permanent hypoparathyroidism [62]. The particles can be injected prior (one month) to surgery or during surgery into the thyroid. A comparative study found that protection of the parathyroid glands was better after the intratumoral administration prior to surgery [107]. The effect of permanent hypoparathyroidism was not evaluated. When used in endoscopic TC surgery, the average number of lymph nodes removed was higher in interventions for which carbon nanoparticles were used. Incidental parathyroidectomy was lower but the incidence of hypoparathyroidism was not significantly different [108]. It may be suspected that the parathyroid glands possess the potential to compensate for a certain amount of tissue damage. Increased mitotic activity (0.9–1.5%) of the chief cells has been reported after mechanical damage of the parathyroid glands in rats [109].

Thermal ablation for papillary microcarcinoma up to ≤1 cm in size is very common in China. Ablation is either performed by microwave, laser, or radiofrequency exposure, resulting in coagulation necrosis of the tumor cells [110]. Thermal ablation appears to result in satisfactory therapeutic effects with minimal postoperative trauma but bears the risk of damage of the surrounding issue. Carbon nanoparticles may also be used for this indication. When injected into thyroid cancer TPC-1 xenografts in mice, the particles absorbed NIR light, converting light into heat and achieving a temperature of 50–56 °C in the tumor, visualized by IR thermal imaging [63]. With this photothermal therapy, a temperature of 53 °C resulted in complete ablation of the tumor without overt systemic toxicity, as identified by body weight (as the only parameter provided). The use of carbon nanoparticles would act only on the tumor tissue because they are not taken up by the parathyroid glands. Further, the authors claimed that damage of the recurrent laryngeal nerve could be prevented by this technique.

The use of functionalized bio-affinity nanoparticles (BioNanoFluid), which are 0.25–10 µm-long and 25–25 nm-thick carboxyl functionalized carbon nanotubes, sparsely coated with gold, is another option for thermal ablation of TC. The particles were pegylated and functionalized with recombinant TSH, purified human TSH, or anti-TSH antibody. Cytotoxicity of the particles after irradiation with a 532 nm power laser was determined in B-CPAP cells (TSH-receptor positive PTC cell line) and NSC-34 TSH-receptor negative mouse neuroblastoma-motor neuron cells [64]. Only the B-CPAP cells reacted, and the particles coated with recombinant TSH showed the highest efficacy.

### 5.2. Inorganic Nanoparticles: Metal-Containing Nanoparticles

Gold nanoparticles: Treatment with small (9.2 ± 0.6 nm) gold NPs downregulated chaperonin-containing TCP-1 subunit 3 (CCT3), which assists in protein folding, cell proliferation, migration, and invasion in the PTC cells B-CPAP and TPC-1, and decreased the viability by increasing apoptosis [65]. In the nano-assemblies, gold NPs and perfluorohexane were surrounded by a lipid shell and the complex functionalized with cetuximab, a monoclonal antibody for targeting EGFR-expressing tumors, e.g., ATC. LIFUS can increase the efficacy of chemotherapeutic drugs by triggering their release from nanocarriers [111]. Upon LIFUS, the shell ruptured and the payload was released. The delivery of the paclitaxel prodrug CYT-21625 together with tumor necrosis factor alpha (TNF-α) in pegylated gold NPs decreased the tumor burden in mice with metastatic FTC-133 and 8505C xenografts [67]. The mechanism by which hyaluronic acid- and oleic acid-coated gold nanoparticles functionalized with ATC-specific ligands, such as holo-transferrin-, EGF-, or lapatinib acting more cytotoxic on human TC 8505C cells than on HaCaT keratinocytes, is by absorption of NIR and conversion to thermal energy (photothermal therapy) [68]. Targeting of the EGFR with EGF or lapatinib acted less potently than the holo-transferrin coating. The relatively large (300–670 nm) particles were safe and accumulated mainly in the liver. Anti-tumor action in vivo was not studied.

Production of 20 nm silver NPs by green synthesis using plants and their extracts to avoid hazardous byproducts were reported to be cytotoxic to human TC SW579 cells and 70 nm silver NPs biosynthesized with cell-free culture filtrate of *Pseudomonas aeruginosa* as the reducing mediator of AgNO_3_ decreased viability in TPC-1 cells [69,70]. Because silver NPs are cytotoxic for a broad panel of cells, it would have been essential to test normal cells in parallel.

Double tumor-targeting by acidic pH and by transferrin receptor was expected to increase delivery to PTC by heptapeptide-functionalized, silk fibroin-coated selenium NPs loaded with the anti-tumor agent fingolimod [66]. The particles showed tumor cell-specific cytotoxicity and reduced xenograft growth of K1 cells derived from PTC in nude mice.

### 5.3. Other Inorganic Materials

Doxorubicin can be incorporated into NPs to decrease its irreversible cardiotoxicity and reversible nephrotoxicity with pegylated (Doxil^®^, Lipodox^®^) and unpegylated (Myocet^®^) liposomes as preferred carriers. However, also pegylated doxorubicin-loaded kaolinite nanoclay may be suitable because it is more cytotoxic to TPC-1 cells than the free drug [71]. Coating with potassium iodide as a substrate for the sodium-iodide symporter (NIS) was used for targeting. The 150–200 nm KI@doxorubicin-kaolin_MeOH_ NPs passively target NIS in papillary thyroid cancer TPC-1 cells in minipigs upon intratumoral injection. When administered intravenously to rabbits fed an iodine-free diet, the NPs targeted tumor tissue actively. This approach may be suitable for the treatment of NIS-expressing TC.

### 5.4. Inorganic and Hydrid Nanoparticles: Mesoporous Silica and Organic Mesoporous Silica Nanoparticles (MSNs)

MSNs are often used as nanocarriers because they possess excellent physicochemical stability, a high surface area, non-toxic nature, good surface permeability, and a chemically modifiable surface. Silicon dioxide NPs loaded with doxorubicin and functionalized with TSH for thyroid targeting induced apoptosis in human FTC-133 cells in vitro and reduced xenograft growth in nude mice [72]. The particles had an acid-labile linker to release doxorubicin in the acidic tumor environment. After intravenous injection, the particles led to decreased tumor growth compared to the free drug and prevented cardiotoxicity. Targeting with TSH improved the anti-tumor effect but had no influence on the creatinine kinase levels as an indicator for cardiotoxicity.

MSNs loaded with the heat shock protein 90 inhibitor 17—allylamino-17 demethoxygeldanamycin (17-AAG, tanespimycin) and the mTOR inhibitor Torin 2 9-(6-aminopyridin-3-yl)-1-[3-(trifluoromethyl)phenyl]-1H,2H-benzo[h] 1,6-naphthyridin-2-one) target the VEGFR2, which plays a critical role in transforming precancerous lesions to malignant tumors. The NPs inhibited growth of human anaplastic carcinoma FRO cells more effectively compared with each single agent and did not affect Nthy-ori3–1 normal human cells [73]. The particles were administered into the tumor tissue and may represent an option for treatment of inoperable primary tumors. Transferrin-coated mesoporous silica particles co-loaded with the multi-kinase inhibitor sorafenib induced apoptosis in radioiodine-resistant TCP-1 and B-CPAP cells prepared by culturing with increasing sublethal ^131^I concentrations for several generations [74]. Encapsulation could improve the low oral availability and poor water solubility, light sensitivity, and high off-target toxicity of the drug. The action of the transferrin-coated NPs was much better than the action of the non-targeted ones in terms of downregulating the MAPK/RAS/RAF/MEK/ERK pathway.

Mesoporous organic silica is a type of silica-containing organic group that gives rise to mesoporosity. Such MSNs loaded with doxorubicin were more successful than the melanin-linked NPs produced from dopamine hydrochloride. While the melanin-linked NPs had a doxorubicin loading capacity of 20%, the mesoporous organic silica particles reached 47.02% [75,76]. Coating MSNs with bovine serum albumin stabilized the doxorubicin loading and induced uptake by drug-sensitive anaplastic thyroid cancer HTh74 cells but not by human HaCaT keratinocytes. These NPs could revert drug resistance by reducing the efflux of doxorubicin.

### 5.5. Organic Nanoparticles: Lipid Nanoparticles

Liposomes are one of the most established nanocarriers. They allow delivery of various drugs with good encapsulation capacity and prolonged circulation, such as in daunorubicin and pegylated liposomal doxorubicin. Further, they enable functionalization with different targeting molecules and are biocompatible. The nucleoside analog gemcitabine has various limitations. It is quickly metabolized and excreted, and causes various side effects and resistance in tumor cells. The hydrophilic gemcitabine molecule is difficult to incorporate into most nanocarriers. Gemcitabine-loaded liposomes were markedly more cytotoxic to ARO cells, derived from an ATC, than the free drug, but in the absence of comparison to other cells, the advantage of encapsulation was not clear [77]. In the reported formulation, ammonium sulphate in the internal compartments of liposomes elicited the protonation of gemcitabine and reduced drug back-diffusion from the liposomes and had an encapsulation efficacy of ~90%. Because gemcitabine administration is complicated by hematological and other toxicities, the assessment of biocompatibility of the formulation is essential.

Increased antiproliferative efficacy was shown for liposomes loaded with all-trans retinoic acid in comparison with the free drug on three different human thyroid carcinoma cell lines (PTC-1, B-CPAP, and FRO) but its action on normal cells was not studied [78]. Lipid polymer NPs loaded with cisplatin were developed and TSH introduced for tumor targeting [112]. A 4-fold higher accumulation of the TSH-conjugated NPs was observed compared to the non-targeted NPs in FTC 133 xenografts. The TSH-conjugated nanoparticles showed a significantly enhanced anticancer effect compared to the other groups. Similarly, 3.5 times more TSH-targeted gemcitabine-loaded liposomes accumulated in xenografts of CHO cells transfected with TSHR, and their anti-tumor action in FTC-133 xenografts was significantly higher than that of not targeted liposomes [113].

microRNA-34b-5p-loaded pegylated lipid particles were tested in human ATC cells (8505C and BHT-101) and in immortalized follicular epithelial cNthy-ori 3-1 cells as controls [79]. miR-34b inhibited cancer cell proliferation, migration, and angiogenesis in vitro and reduced the size of the BHT-101 xenografts upon intravenous injection into nude mice.

### 5.6. Organic Nanoparticles: Polymers

Pegylated PLGA NPs were used to deliver a combination of sorafenib and all-trans retinoic acid as differentiating-promoting drugs into FTC133 cells and their xenografts in a BALB/c nude mouse model. Their intravenous injection resulted in lower toxicity than sorafenib that had not been encapsulated, and the expression of NIS and Tg increased as an indication of re-differentiation by retinoic acid [80]. Resveratrol, a polyphenol from plants such as grapes, induces differentiation and apoptosis of ATC cells. However, its action is limited by its rapid transformation and elimination. When loaded into pegylated polycaprolactone (PCL_4k_) particles coated with nonapeptide PEP-1 to target the IL-13 receptor alpha 2, over-expressed on tumor cells, e.g., the ATC cell line THJ-16T, resveratrol-treated tumors were similar in size to docetaxel- and doxorubicin-treated tumors [81].

Chitosan-modified PLGA nanoparticles: siRNA to telomerase reverse transcriptase (TERT) was encapsulated in this type of NP in order to overcome drug bolus release. These NPs retarded the growth of ATC cells (8505C and CAL-62) and of their xenografts in SCID mice without systemic toxicity. Telomere lengths were not affected by the treatment [82]. Another nanoplatform for systemic siRNA delivery is poly[2,6-(4,4-bis-(2-ethylhexyl)-4H-cyclopenta [2,1-b;3,4-b′]dithiophene)-alt-4,7 (2,1,3-benzothiadiazole)] (PolyPCPDTBT) + amphiphilic cationic lipid for complexing siRNA aggregated by self-assembly [61]. The encapsulation rate of BRAF siRNA was 50%. Particles were coated with 1,2-distearoyl-sn-glycero-3- phosphoethanolamine-N-[methoxy(polyethylene glycol)-2000] and used in an orthotopic ATC model with 8505C cells. This treatment did not completely inhibit metastasis but silencing of BRAF and inhibition of proliferation in the absence of systemic toxicity was reported.

### 5.7. Other Organic Materials

Gemcitabine plus linoleic acid self-assembles to NPs of ~100 nm. These particles induced apoptosis in follicular thyroid carcinoma FTC133 and in papillary thyroid carcinoma B-CPAP cells. Because the nanoformulations were predicted to damage red blood cells, hemolysis could be excluded in hemolysis assays [83].

Nanobubbles are tiny gas-filled cavities having unique physical characteristics. Perfluoropentane-cored glycol chitosan nanobubbles loaded with doxorubicin enhanced cytotoxicity on ATC-derived CAL-62 cells [84]. The bubbles were bigger than NPs normally used for targeted delivery (356.2 ± 15.1 nm). Upon extracorporeal shock wave application (0.59 mJ/mm^2^, 500 pulses), doxorubicin-release was induced. Human ATC xenograft tumors were smaller and contained more doxorubicin compared to the free drug. No cardiotoxicity was observed.

Overall, inorganic and organic NPs have been studied in TC. Gold NPs and mesoporous (hybrid) silica NPs were reported to be suitable for delivery of conventional drugs and proteins. Gold particles may be also used for photothermal therapy. Targeting strategies range from general principles in cancer treatment (pH-dependency, overexpression of transferrin or VEGF receptor) to more TC-specific targets, such as the TSH receptor. The latter strategy has the disadvantage that de-differentiated TC usually do not express the receptor any more. Liposomes and polymeric NPs were used for delivery of conventional drugs, re-differentiating compounds, and siRNA. Active and passive targeting was effective.

Inorganic and organic NPs possess different advantages and limitations. The inorganic NPs, e.g., gold NPs, enable the use of additional technologies but could pose problems, for example, regarding accumulation in the human body. Organic NPs, typically lipid nanoparticles, are faster degraded and removed from the body. They offer the possibility to load a broad variety of payloads but the option to use additional anti-tumor strategies (e.g., photodynamic therapy and hyperthermia) is more limited. Reproducible, large-scale synthesis represents a challenge for all NP-based treatments. More information on nanomedicine in cancer is available in reviews on this topic (e.g., [114,115]).

### 5.8. NPs in Clinical Trials

Despite the various proposed uses of NPs in diagnosis and treatment of TC, data from clinical trials are limited. Table 2 summarizes clinical trials of NPs in TC.

CALAA-01 is a transferrin-targeted nanocomplex consisting of siRNA to reduce expression of the M2 subunit of ribonucleotide reductase and cyclodextrin-containing polymer. The trial was terminated due to dose-limiting toxicities (e.g., grade 3 ischemic colitis, grade 2 diarrhea, and grade 1 fever, as well as grade 4 fatigue, grade 2 flu-like symptoms, grade 2 muscular spasm, and grade 1 nausea).

Future and ongoing trials will evaluate the use of several nanoparticles as a tracer for lymph node mapping to improve the surgical treatment of thyroid cancer. These particles include carbon nanoparticles alone, carbon nanoparticles in combination with indocyanine green dye, and anti-Tg antibody functionalized fluorescent polystyrene particles [117].

For multidrug chemotherapy of TC, the efficacy of the approved albumin-bound particle form of paclitaxel (nab-paclitaxel, Abraxane^®^) is being evaluated in combination with the anti-PDL-1 antibody Atezolizumab (NCT03181100, phase II) and in combination with the glycogen synthase kinase 3 beta (GSK-3β) inhibitor, 9-ING-41 (NCT03678883, phase I/II).

## 6. Conclusions

Compared to other tumors, a relatively low number of NPs has been studied for diagnosis and treatment of TC. Reasons for that may be that TC is not a common cancer and TC, with the exception of de-differentiated tumors and ATC, is not highly aggressive. Tumor accumulation of NPs can either use passive targeting by the EPR effect or use tumor-specific targeting molecules. NPs could improve diagnosis and monitoring of TC by more sensitive sensor technologies, e.g., BRAF V600 mutation and for imaging. Encapsulation into nanocarriers increased the efficacy of doxorubicin, sorafenib, and gemcitabine treatment, and decreased their adverse effects. Improved delivery of retinoic acid to TC cells could improve the re-differentiation therapy of de-differentiated TC.

All NPs, except carbon NPs during TC surgery, are in the pre-clinical phase of drug development and, quite frequently, only in vitro cellular studies, often without comparison to normal cells, are available. Although encouraging results from animal studies have been reported, their value for predicting efficacy in humans is subject to the inherent limitations of the models. These include the clonal origin of the xenografts, leading to lower heterogeneity, the larger relative size of the xenografts compared to human tumors but smaller size at treatment, immunodeficient status of the athymic mice, inoculation at a young age, subcutaneous versus orthotopic localization, and often the absence of metastases in the mouse models [37]. These differences may not be very relevant because rapid growth and high vascularization are also seen in human ATC. Further, metastatic disease can be mimicked by the generation of orthotopic tumors using injection of the appropriate cells (e.g., 8505C) into the thyroid. However, human tumors still differ from the xenografts by their smaller sizes relative to body weight, and the older age of the host. The benefit of using carbon NPs for improved TC surgery is still not clear. Specific technologies less suitable for the treatment of deeply located cancers have some potential for unresectable ATC, namely LIFUS and phototherapy using NIR irradiation.

## Figures and Tables

**Table 1:** Use of nanoparticles (NPs) in preclinical studies. Abbreviations: BRAF, V-raf murine sarcoma viral oncogene homolog B; CT, computed tomography; EGF, epithelial growth factor; LIFUS, low-intensity focused ultrasound; LOD, limit of detection; MSNs, mesoporous silica NPs; NIR, near-infrared; NIS, sodium iodide symporter; PLGA, polylactic-co-glycolid acid; PolyPCPDTBT, poly [2,6-(4,4-bis-(2-ethylhexyl)-4H-cyclopenta [2,1-b;3,4-b′]dithiophene)-alt-4,7(2,1,3-benzothiadiazole)]; Tg, thyroglobulin; SPECT, single photon emission computed tomography; TSH, thyroid stimulating hormone; VEGF, vascular endothelial growth factor.

Application	Nanomaterial	Results	Reference	
SENSORS				
TSH detection	Anti-TSH antibody-conjugated horseradish peroxidase immobilized on platinum NPs	This assay was three times faster and 100 times more sensitive than commercially available assays		[50]
TSH detection	Polyamidoamine dendrimers enlarged binding sites on gold electrodes	LOD of 0.026 mIU/L		[51]
Tg detection	Fluoroimmunodiagnostic nanoplatform using tannylated ferritin nanocages	LOD of 4.3 pg/mL in artificial human serum medium		[52]
Calcitonin detection	Flower-like thin film gold nanoparticles doped in a sol–gel/polyethylene glycol mold	LOD of 0.707 pg/mL		[53]
Calcitonin detection	Gold nanoparticles and graphene oxide on the activated surface of a glassy carbon electrode	LOD of 0.7 pg/mL		[54]
BRAF mutation	Gold amplified biosensor	LOD of 0.35 amol/L		[55]
BRAF mutation	S-regulated boron nitride quantum dots	LOD of 0.3 pmol/L		[56]
IMAGING				
Tumor detection	Iodinated gold nanoclusters synthesized via bovine serum albumin and chloramine-T	Detection of tumors ≥ two mm^2^ in mice by combined CT/NIR imaging		[57]
	^131^I-VEGF-targeted mesoporous silica particles	Strong SPECT signal only after intratumoral injection		[58]
	Phase-changeable PLGA polymeric nanoparticles decorated with SHP2 antibody	Strong label of tumor tissue upon irradiation with LIFUS in tumor-bearing mice		[59]
	[^64^Cu] CuS NPs	Localization of the tumor in mice and reduction by 83% by NIR irradiation		[60]
	BRAF silencing siRNA-loaded poly [2,6-(4,4-bis-(2-ethylhexyl)-4H-cyclopenta [2,1-b;3,4-b′]dithiophene)-alt-4,7(2,1,3-benzothiadiazole)] NPs	Good localization of BRAF V600E-mutated 8505C cells in mice by NIR imaging		[61]
TREATMENT				
Improved surgery	Carbon NPs	More lymph nodes identified; no decrease in the rate of permanent hypoparathyroidism		[62]
Thermal ablation	Carbon NPs for thermal ablation of microcarcinoma	Thermal ablation of TPC-1 xenografts in mice after NIR irradiation		[63]
Thermal ablation	Carbon nanotubes (BioNanoFluid) functionalized with recombinant TSH	Selective death of TSH-receptor positive B-CPAP cells		[64]
Anti-tumor action of NPs	Gold NPs	Decrease of cell proliferation, migration and invasion in the PTC cells B-CPAP and TPC-1 and increase of apoptosis		[65]
Improved delivery	Heptapeptide-functionalized, silk fibroin coated selenium NPs loaded with the anti-tumor agent fingolimod	Tumor cell-specific cytotoxicity and reduced tumor size of K1 xenografts in mice.		[66]
Improved delivery	Pegylated gold NPs with paclitaxel prodrug CYT-21625 and tumor necrosis factor alpha	Decreased tumor burden in mice with metastatic FTC-133 and 8505C xenografts		[67]
Photothermal therapy	Hyaluronic acid- and oleic acid-coated gold NPs functionalized with holo-transferrin-, EGF- or lapatinib	Selective cytotoxicity in human TC 8505C cells after NIR irradiation; best efficacy by transferrin coating		[68]
Anti-tumor action of NPs	Silver NPs (20 nm and 70 nm)	Cytotoxicity in SW579 cells and TPC-1 cells but tumor cells specificity unclear		[69,70]
Improved delivery	KI@doxorubicin-kaolin_MeOH_ NPs	Targeting of TPC-1 cells in minipigs upon intratumoral injection		[71]
Improved delivery	Silicon dioxide NPs loaded with doxorubicin and functionalized with TSH	Reduced growth of FTC-133 xenograft in mice in the absence of cardiotoxicity		[72]
Improved delivery	MSNs loaded with tanespimycin and Torin 2	Selective growth inhibition of FRO cells		[73]
Improved delivery	Transferrin-coated MSNs loaded with the multi-kinase inhibitor sorafenib	Induction of apoptosis in radioiodine-resistant TCP-1 and B-CPAP cells		[74]
Improved delivery	Bovine serum albumin coated MSNs loaded with doxorubicin	Selective uptake by HTh74 cells		[75,76]
Improved delivery	Gemcitabine-loaded liposomes	Higher cytotoxicity in ARO cells; tumor selectivity not studied		[77]
Improved delivery	Liposomes loaded with all-trans retinoic acid	Higher cytotoxicity in ARO cells; tumor selectivity not studied		[78]
Improved delivery	microRNA-34b-5p-loaded pegylated lipid NPs	Reduction of BHT-101 xenograft growth in mice.		[79]
Improved delivery	Pegylated PLGA NPs loaded with sorafenib and all-trans retinoic acid	Increased NIS and Tg expression of FTC133 xenografts in mice with reduced general toxicity		[80]
Improved delivery	Pegylated polycaprolactone (PCL4k) particles coated with nonapeptide PEP-1 and loaded with resveratrol	Similar efficacy to docetaxel and doxorubicin in THJ-16T xenografts in mice		[81]
Improved delivery	siRNA to telomerase reverse transcriptase encapsulated in chitosan-modified PLGA NPs	Growth inhibition of 8505C and CAL-62 xenografts in mice		[82]
Improved delivery	Poly PCPDTBT + BRAF siRNA complexes coated with 1,2-distearoyl-sn-glycero-3- phosphoethanolamine-N-[methoxy(polyethylene glycol)-2000]	Growth inhibition of 8505C xenografts in mice		[61]
Anti-tumor action of NPs	Gemcitabine + linoleic acid complexes	Induction of apoptosis in FTC133 and B-CPAP cells		[83]
Improved delivery	Perfluoropentane-cored glycol chitosan nanobubbles loaded with doxorubicin	Reduction of CAL-62 xenografts after extracorporeal shock wave in mice		[84]

**Table 2:** Overview of clinical trials assessing the use of nanoparticles in TC diagnosis and treatment. Abbreviation: DLT, dose-limiting toxicity.

Trial Number	Title	Phase	Status	Start
DIAGNOSIS				
ChiCTR2000036620	Study on the clinical value of rapid detection of thyroglobulin by nano-fluorescent microsphere method	N/A	Not yet recruiting	January 2020
ChiCTR1900027016	A prospective study for nano carbon combined with fluorescence tracing in sentinel lymph node biopsy of thyroid papillary carcinoma	N/A	Not yet recruiting	December 2019
TREATMENT				
NCT04312087	Lateral Neck Lymph Node Mapping in Thyroid Cancer	N/A	Recruiting	August 2016
NCT02724176	Potential Role for Carbon Nanoparticles to Guide Central Neck Dissection in Patients With Papillary Thyroid Cancer	N/A	* Results published	January 2012
ChiCTR-TRC-13003404	Image contrast and protection of carbon nanoparticles on parathyroid	II	No results published	June 2013
NCT01927887	Pre-Operative Nodal Staging of Thyroid Cancer Using USPIO MRI: Preliminary Study	N/A	12 patients enrolled	July 2012
NCT00689065	Safety Study of CALAA-01 to Treat Solid Tumor Cancers	I	DLT occurred	May 2008

* More lymph nodes identified; at Day 1 but not Day 7 lower incidence of hypoparathyroidism [116].

## Abbreviations

17-AAG	17-allylamino-17 demethoxygeldamycin
ATC	anaplastic thyroid carcinoma
BRAF	V-raf murine sarcoma viral oncogene homolog B
CCT3	chaperonin-containing TCP-1 subunit 3
CT	computed tomography
DLS	dynamic light scattering
DTC	differentiated thyroid cancer
EBRT	external beam radiotherapy
EGFR	epidermal growth factor receptor
ECL	electrochemoluminescence
EpCAM	epithelial cell adhesion molecule
EPR	enhanced penetration and retention
ERK	extracellular signal-regulated kinase
ESF	European Science Foundation
ESMO	European Society of Medical Oncology
FDA	Food and Drug Agency
FDG	[^18^F]fluorodeoxyglucose
FGFR	fibroblast growth factor receptor
FITC	fluorescein 5-isothiocyanate
FLT3	FMS-like tyrosine kinase -3
FRET	fluorescence energy transfer
FTC	follicular thyroid carcinoma
GFR	growth factor receptors
GSK-3β	glycogen synthase kinase 3 beta
HRP	horseradish peroxidase
IGF2BP1	IGF2 mRNA binding protein 1
LIFUS	low intensity focused ultrasound
LOD	limit of detection
LSPR	localized surface plasmon resonance
MALDI-TOF	matrix-assisted laser desorption ionization-time of flight
MAGEA3	melanoma-associated antigen A3
MAPK	mitogen-activated protein kinase
MEK	mitogen-activated protein kinase kinase
MMP	matrix metalloprotease
MPS	mononuclear phagocyte system
MRI	magnetic resonance imaging
MSNs	mesoporous silica NPs
MTC	medullary thyroid carcinoma
Nab-PTX	nanoalbumin-bound paclitaxel
NETs	neuroendocrine tumors
NIH	United States’ National Institutes of Health
NIR	near-infrared
NIS	sodium iodide symporter
NP	nanoparticle
PCK4k	polycaprolactone
PCR	polymerase chain reaction
PDGFR	platelet-derived growth factor receptor
PDK	3-phosphoinositide-dependent kinase
PEG	polyethylene glycol
PET	positron emission tomography
PI3K	phosphatidylinositol 3-kinase
PLG	polylactic-co-glycolid acid
PolyPCPDTBT	poly[2,6-(4,4-bis-(2-ethylhexyl)-4H-cyclopenta [2,1-b;3,4-b′]dithiophene)-alt-4,7(2,1,3-benzothiadiazole)]
Pt	platinum
PTC	papillary thyroid carcinoma
PTEN	phosphatase and tensin homolog deleted on chromosome 10
RAS	rat sarcoma virus
RTK	receptor tyrosine kinases
SERS	surface enhanced Raman scattering
SPARC	secreted protein acidic and rich in cysteine
SPECT	single photon emission computed tomography
SWSV	Square wave stripping voltammetry
TC	thyroid cancer
TERT	telomerase reverse transcriptase
Tg	thyroglobulin
TgAb	thyroglobulin antibodies
TKIs	tyrosine kinase inhibitors
TNF-α	tumor necrosis factor alpha
TSH	thyroid stimulating hormone
VCAM-1	vascular cell adhesion molecule 1
VEGFR	vascular endothelial growth factor receptor
WBC	whole body scan
WHO	World Health Organization

## References

[1] Sung H., Ferlay J., Siegel R.L., Laversanne M., Soerjomataram I., Jemal A., Bray F. (2021). Global Cancer Statistics 2020: GLOBOCAN Estimates of Incidence and Mortality Worldwide for 36 Cancers in 185 Countries. CA Cancer J. Clin..

[2] van der Zwan J.M., Mallone S., van Dijk B., Bielska-Lasota M., Otter R., Foschi R., Baudin E., Links T.P. (2012). Carcinoma of endocrine organs: Results of the RARECARE project. Eur. J. Cancer.

[3] Thomas F., Nesse R.M., Gatenby R., Gidoin C., Renaud F., Roche B., Ujvari B. (2016). Evolutionary Ecology of Organs: A Missing Link in Cancer Development?. Trends Cancer.

[4] Davies J.A. (2004). Inverse Correlation Between an Organ’s Cancer Rate and Its Evolutionary Antiquity. Organogenesis.

[5] Tomasetti C., Vogelstein B. (2015). Cancer etiology. Variation in cancer risk among tissues can be explained by the number of stem cell divisions. Science.

[6] López-Lázaro M. (2018). Cancer etiology: Variation in cancer risk among tissues is poorly explained by the number of gene mutations. Genes Chromosomes Cancer.

[7] Angelousi A., Alexandraki K.I., Kyriakopoulos G., Tsoli M., Thomas D., Kaltsas G., Grossman A. (2020). Neoplastic metastases to the endocrine glands. Endocr. Relat. Cancer.

[8] Sharma P., Johns M., Anderson K., Meyers A. (2020). Thyroid Cancer: Practice Essentials, Overview, Clinical Presentation. Thyroid Cancer.

[9] Amaral M., Afonso R.A., Gaspar M.M., Reis C.P. (2020). Anaplastic thyroid cancer: How far can we go?. Excli J..

[10] Soares P., Póvoa A.A., Melo M., Vinagre J., Máximo V., Eloy C., Cameselle-Teijeiro J.M., Sobrinho-Simões M. (2021). Molecular Pathology of Non-familial Follicular Epithelial-Derived Thyroid Cancer in Adults: From RAS/BRAF-like Tumor Designations to Molecular Risk Stratification. Endocr. Pathol..

[11] Xu S., Han Y. (2021). The Overdiagnosis of Thyroid Micropapillary Carcinoma: The Rising Incidence, Inert Biological Behavior, and Countermeasures. J. Oncol..

[12] Kaltsas G., Androulakis I.I., de Herder W.W., Grossman A.B. (2010). Paraneoplastic syndromes secondary to neuroendocrine tumours. Endocr. Relat. Cancer.

[13] Oronsky B., Ma P.C., Morgensztern D., Carter C.A. (2017). Nothing But NET: A Review of Neuroendocrine Tumors and Carcinomas. Neoplasia.

[14] Clayman G. Thyroid Cancer Symptoms, Diagnosis, and Treatments. https://www.endocrineweb.com/conditions/thyroid-cancer/thyroid-cancer.

[15] Skwiersky S., Hevroni G., Singh G., Hope L., Haidary T., Salifu M.O., McFarlane S.I. (2020). Concurrent Anaplastic and Papillary Thyroid Carcinomas: A Case Report. Am. J. Med. Case Rep..

[16] Agrawal M., Uppin S.G., Challa S., Prayaga A.K. (2013). Carcinosarcoma thyroid: An unusual morphology with a review of the literature. South Asian J. Cancer.

[17] Bible K.C., Kebebew E., Brierley J., Brito J.P., Cabanillas M.E., Clark T.J., Di Cristofano A., Foote R., Giordano T., Kasperbauer J. (2021). 2021 American Thyroid Association Guidelines for Management of Patients with Anaplastic Thyroid Cancer. Thyroid.

[18] Filetti S., Durante C., Hartl D., Leboulleux S., Locati L.D., Newbold K., Papotti M.G., Berruti A. (2019). Thyroid cancer: ESMO Clinical Practice Guidelines for diagnosis, treatment and follow-up†. Ann. Oncol..

[19] Tuttle R.M., Ahuja S., Avram A.M., Bernet V.J., Bourguet P., Daniels G.H., Dillehay G., Draganescu C., Flux G., Führer D. (2019). Controversies, Consensus, and Collaboration in the Use of (131)I Therapy in Differentiated Thyroid Cancer: A Joint Statement from the American Thyroid Association, the European Association of Nuclear Medicine, the Society of Nuclear Medicine and Molecular Imaging, and the European Thyroid Association. Thyroid.

[20] Gild M.L., Tsang V.H.M., Clifton-Bligh R.J., Robinson B.G. (2021). Multikinase inhibitors in thyroid cancer: Timing of targeted therapy. Nat. Rev. Endocrinol..

[21] Tolcher A.W., Peng W., Calvo E. (2018). Rational Approaches for Combination Therapy Strategies Targeting the MAP Kinase Pathway in Solid Tumors. Mol. Cancer Ther..

[22] Fleeman N., Houten R., Chaplin M., Beale S., Boland A., Dundar Y., Greenhalgh J., Duarte R., Shenoy A. (2019). A systematic review of lenvatinib and sorafenib for treating progressive, locally advanced or metastatic, differentiated thyroid cancer after treatment with radioactive iodine. BMC Cancer.

[23] Koehler V.F., Adam P., Frank-Raue K., Raue F., Berg E., Hoster E., Allelein S., Schott M., Kroiss M., Spitzweg C. (2021). Real-World Efficacy and Safety of Cabozantinib and Vandetanib in Advanced Medullary Thyroid Cancer. Thyroid.

[24] Khunger A., Khunger M., Velcheti V. (2018). Dabrafenib in combination with trametinib in the treatment of patients with BRAF V600-positive advanced or metastatic non-small cell lung cancer: Clinical evidence and experience. Ther. Adv. Respir. Dis..

[25] Subbiah V., Kreitman R.J., Wainberg Z.A., Cho J.Y., Schellens J.H.M., Soria J.C., Wen P.Y., Zielinski C., Cabanillas M.E., Urbanowitz G. (2018). Dabrafenib and Trametinib Treatment in Patients with Locally Advanced or Metastatic BRAF V600-Mutant Anaplastic Thyroid Cancer. J. Clin. Oncol..

[26] Cabanillas M.E., Habra M.A. (2016). Lenvatinib: Role in thyroid cancer and other solid tumors. Cancer Treat. Rev..

[27] De Leo S., Trevisan M., Fugazzola L. (2020). Recent advances in the management of anaplastic thyroid cancer. Thyroid Res..

[28] Webster T.J. (2006). Nanomedicine: What’s in a definition?. Int. J. Nanomed..

[29] Martinelli C., Pucci C., Ciofani G. (2019). Nanostructured carriers as innovative tools for cancer diagnosis and therapy. APL Bioeng..

[30] Navya P.N., Kaphle A., Srinivas S.P., Bhargava S.K., Rotello V.M., Daima H.K. (2019). Current trends and challenges in cancer management and therapy using designer nanomaterials. Nano Converg..

[31] Yao Y., Zhou Y., Liu L., Xu Y., Chen Q., Wang Y., Wu S., Deng Y., Zhang J., Shao A. (2020). Nanoparticle-Based Drug Delivery in Cancer Therapy and Its Role in Overcoming Drug Resistance. Front. Mol. Biosci..

[32] Huang X., O’Connor R., Kwizera E.A. (2017). Gold Nanoparticle Based Platforms for Circulating Cancer Marker Detection. Nanotheranostics.

[33] Fröhlich E. (2012). The role of surface charge in cellular uptake and cytotoxicity of medical nanoparticles. Int. J. Nanomed..

[34] Zein R., Sharrouf W., Selting K. (2020). Physical Properties of Nanoparticles That Result in Improved Cancer Targeting. J. Oncol..

[35] Huynh E., Zheng G. (2015). Cancer nanomedicine: Addressing the dark side of the enhanced permeability and retention effect. Nanomedicine (London, England).

[36] Thomas O.S., Weber W. (2019). Overcoming Physiological Barriers to Nanoparticle Delivery-Are We There Yet?. Front. Bioeng. Biotechnol..

[37] Golombek S.K., May J.N., Theek B., Appold L., Drude N., Kiessling F., Lammers T. (2018). Tumor targeting via EPR: Strategies to enhance patient responses. Adv. Drug Deliv. Rev..

[38] Lee H., Gaddy D., Ventura M., Bernards N., de Souza R., Kirpotin D., Wickham T., Fitzgerald J., Zheng J., Hendriks B.S. (2018). Companion Diagnostic (64)Cu-Liposome Positron Emission Tomography Enables Characterization of Drug Delivery to Tumors and Predicts Response to Cancer Nanomedicines. Theranostics.

[39] Wilhelm S., Tavares A., Dai Q., Ohta S., Audet J., Dvorak H., Chan W. (2016). Analysis of nanoparticle delivery to tumours. Nat. Rev. Mater..

[40] van Vlerken L.E., Duan Z., Little S.R., Seiden M.V., Amiji M.M. (2008). Biodistribution and pharmacokinetic analysis of Paclitaxel and ceramide administered in multifunctional polymer-blend nanoparticles in drug resistant breast cancer model. Mol. Pharm..

[41] Rosenblum D., Joshi N., Tao W., Karp J.M., Peer D. (2018). Progress and challenges towards targeted delivery of cancer therapeutics. Nat. Commun..

[42] Schmidt M.M., Wittrup K.D. (2009). A modeling analysis of the effects of molecular size and binding affinity on tumor targeting. Mol. Cancer Ther..

[43] Yameen B., Choi W.I., Vilos C., Swami A., Shi J., Farokhzad O.C. (2014). Insight into nanoparticle cellular uptake and intracellular targeting. J. Control. Release.

[44] Sahay G., Querbes W., Alabi C., Eltoukhy A., Sarkar S., Zurenko C., Karagiannis E., Love K., Chen D., Zoncu R. (2013). Efficiency of siRNA delivery by lipid nanoparticles is limited by endocytic recycling. Nat. Biotechnol..

[45] Fröhlich E. (2016). Cellular elimination of nanoparticles. Environ. Toxicol. Pharmacol..

[46] Fornaguera C., García-Celma M.J. (2017). Personalized Nanomedicine: A Revolution at the Nanoscale. J. Pers. Med..

[47] Huang B., Abraham W.D., Zheng Y., Bustamante López S.C., Luo S.S., Irvine D.J. (2015). Active targeting of chemotherapy to disseminated tumors using nanoparticle-carrying T cells. Sci. Transl. Med..

[48] Zocchi M.R., Tosetti F., Benelli R., Poggi A. (2020). Cancer Nanomedicine Special Issue Review Anticancer Drug Delivery with Nanoparticles: Extracellular Vesicles or Synthetic Nanobeads as Therapeutic Tools for Conventional Treatment or Immunotherapy. Cancers.

[49] Wenande E., Garvey L.H. (2016). Immediate-type hypersensitivity to polyethylene glycols: A review. Clin. Exp. Allergy.

[50] Choi G., Kim E., Park E., Lee J.H. (2017). A cost-effective chemiluminescent biosensor capable of early diagnosing cancer using a combination of magnetic beads and platinum nanoparticles. Talanta.

[51] Ozcan H.M., Aydin U.D. (2021). A simple immunosensor for thyroid stimulating hormone. Artif. Cells Nanomed. Biotechnol..

[52] Turan E., Şahin F., Suludere Z., Tümtürk H. (2019). A fluoroimmunodiagnostic nanoplatform for thyroglobulin detection based on fluorescence quenching signal. Sensor. Actuat. B Chem..

[53] Omer W.E., El-Kemary M.A., Elsaady M.M., Abou-Omar M.N., Youssef A.O., Sayqal A.A., Gouda A.A., Attia M.S. (2020). Highly Efficient Gold Nano-Flower Optical Biosensor Doped in a Sol-Gel/PEG Matrix for the Determination of a Calcitonin Biomarker in Different Serum Samples. ACS Omega.

[54] Alarfaja N., El-Tohamy M. (2017). A label-free electrochemical immunosensor based on gold nanoparticles and graphene oxide for the detection of tumor marker calcitonin. New J. Chem..

[55] Liao K.T., Cheng J.T., Li C.L., Liu R.T., Huang H.J. (2009). Ultra-sensitive detection of mutated papillary thyroid carcinoma DNA using square wave stripping voltammetry method and amplified gold nanoparticle biomarkers. Biosens. Bioelectron..

[56] Liu Y., Wang M., Nie Y., Zhang Q., Ma Q. (2019). Sulfur Regulated Boron Nitride Quantum Dots Electrochemiluminescence with Amplified Surface Plasmon Coupling Strategy for BRAF Gene Detection. Anal. Chem..

[57] Chen X., Zhu H., Huang X., Wang P., Zhang F., Li W., Chen G., Chen B. (2017). Novel iodinated gold nanoclusters for precise diagnosis of thyroid cancer. Nanoscale Res. Lett..

[58] Zhang R., Zhang Y., Tan J., Wang H., Zhang G., Li N., Meng Z., Zhang F., Chang J., Wang R. (2019). Antitumor Effect of (131)I-Labeled Anti-VEGFR2 Targeted Mesoporous Silica Nanoparticles in Anaplastic Thyroid Cancer. Nanoscale Res. Lett..

[59] Hu Z., Yang B., Li T., Li J. (2018). Thyroid Cancer Detection by Ultrasound Molecular Imaging with SHP2-Targeted Perfluorocarbon Nanoparticles. Contrast Media Mol. Imaging.

[60] Zhou M., Chen Y., Adachi M., Wen X., Erwin B., Mawlawi O., Lai S.Y., Li C. (2015). Single agent nanoparticle for radiotherapy and radio-photothermal therapy in anaplastic thyroid cancer. Biomaterials.

[61] Liu Y., Gunda V., Zhu X., Xu X., Wu J., Askhatova D., Farokhzad O.C., Parangi S., Shi J. (2016). Theranostic near-infrared fluorescent nanoplatform for imaging and systemic siRNA delivery to metastatic anaplastic thyroid cancer. Proc. Natl. Acad. Sci. USA.

[62] Liu J., Xu C., Wang R., Han P., Zhao Q., Li H., Bai Y., Liu L., Zhang S., Yao X. (2020). Do carbon nanoparticles really improve thyroid cancer surgery? A retrospective analysis of real-world data. World J. Surg. Oncol..

[63] Huang Y., Zeng G., Xin Q., Yang J., Zeng C., Tang K., Yang S., Tang X. (2020). Carbon nanoparticles suspension injection for photothermal therapy of xenografted human thyroid carcinoma in vivo. MedCom.

[64] Dotan I., Roche P.J., Paliouras M., Mitmaker E.J., Trifiro M.A. (2016). Engineering Multi-Walled Carbon Nanotube Therapeutic Bionanofluids to Selectively Target Papillary Thyroid Cancer Cells. PLoS ONE.

[65] Liu F., Ma D., Chen W., Chen X., Qian Y., Zhao Y., Hu T., Yin R., Zhu Y., Zhang Y. (2019). Gold Nanoparticles Suppressed Proliferation, Migration, and Invasion in Papillary Thyroid Carcinoma Cells via Downregulation of CCT3. J. Nanomater..

[66] Zou X., Jiang Z., Li L., Huang Z. (2021). Selenium nanoparticles coated with pH responsive silk fibroin complex for fingolimod release and enhanced targeting in thyroid cancer. Artif. Cells Nanomed. Biotechnol..

[67] Nilubol N., Yuan Z., Paciotti G.F., Tamarkin L., Sanchez C., Gaskins K., Freedman E.M., Cao S., Zhao J., Kingston D.G.I. (2018). Novel Dual-Action Targeted Nanomedicine in Mice With Metastatic Thyroid Cancer and Pancreatic Neuroendocrine Tumors. J. Natl. Cancer Inst..

[68] Amaral M., Charmier A.J., Afonso R.A., Catarino J., Faísca P., Carvalho L., Ascensão L., Coelho J.M.P., Gaspar M.M., Reis C.P. (2021). Gold-Based Nanoplataform for the Treatment of Anaplastic Thyroid Carcinoma: A Step Forward. Cancers.

[69] Li Y., Wang Y., Cheng B. (2017). In-vitro cytotoxicity of biosynthesized gold nanoparticles against thyroid cancer cell lines. Trop. J. Pharm. Res..

[70] Yang J., Wang Q., Wang C., Yang R., Ahmed M., Kumaran S., Velu P., Li B. (2020). *Pseudomonas aeruginosa* synthesized silver nanoparticles inhibit cell proliferation and induce ROS mediated apoptosis in thyroid cancer cell line (TPC1). Artif. Cells Nanomed. Biotechnol..

[71] Zhang Y., Long M., Huang P., Yang H., Chang S., Hu Y., Tang A., Mao L. (2016). Emerging integrated nanoclay-facilitated drug delivery system for papillary thyroid cancer therapy. Sci. Rep..

[72] Li S., Zhang D., Sheng S., Sun H. (2017). Targeting thyroid cancer with acid-triggered release of doxorubicin from silicon dioxide nanoparticles. Int. J. Nanomed..

[73] Wang C., Zhang R., Tan J., Meng Z., Zhang Y., Li N., Wang H., Chang J., Wang R. (2020). Effect of mesoporous silica nanoparticles co-loading with 17-AAG and Torin2 on anaplastic thyroid carcinoma by targeting VEGFR2. Oncol. Rep..

[74] Ke Y., Xiang C. (2018). Transferrin receptor-targeted HMSN for sorafenib delivery in refractory differentiated thyroid cancer therapy. Int. J. Nanomed..

[75] Wang K., Wang S., Chen K., Zhao Y., Ma X., Wang L. (2018). Doxorubicin-Loaded Melanin Particles for Enhanced Chemotherapy in Drug-Resistant Anaplastic Thyroid Cancer Cells. J. Nanomater..

[76] Han X., Xu X., Tang Y., Zhu F., Tian Y., Liu W., He D., Lu G., Gu Y., Wang S. (2020). BSA-Stabilized Mesoporous Organosilica Nanoparticles Reversed Chemotherapy Resistance of Anaplastic Thyroid Cancer by Increasing Drug Uptake and Reducing Cellular Efflux. Front. Mol. Biosci..

[77] Celano M., Calvagno M.G., Bulotta S., Paolino D., Arturi F., Rotiroti D., Filetti S., Fresta M., Russo D. (2004). Cytotoxic effects of gemcitabine-loaded liposomes in human anaplastic thyroid carcinoma cells. BMC Cancer.

[78] Cristiano M.C., Cosco D., Celia C., Tudose A., Mare R., Paolino D., Fresta M. (2017). Anticancer activity of all-trans retinoic acid-loaded liposomes on human thyroid carcinoma cells. Colloids Surf. B Biointerfaces.

[79] Maroof H., Islam F., Dong L., Ajjikuttira P., Gopalan V., McMillan N.A.J., Lam A.K. (2018). Liposomal Delivery of miR-34b-5p Induced Cancer Cell Death in Thyroid Carcinoma. Cells.

[80] Li S., Dong S., Xu W., Jiang Y., Li Z. (2019). Polymer Nanoformulation of Sorafenib and All-Trans Retinoic Acid for Synergistic Inhibition of Thyroid Cancer. Front. Pharmacol..

[81] Xiong L., Lin X.M., Nie J.H., Ye H.S., Liu J. (2021). Resveratrol and its Nanoparticle suppress Doxorubicin/Docetaxel-resistant anaplastic Thyroid Cancer Cells in vitro and in vivo. Nanotheranostics.

[82] Lombardo G.E., Maggisano V., Celano M., Cosco D., Mignogna C., Baldan F., Lepore S.M., Allegri L., Moretti S., Durante C. (2018). Anti-hTERT siRNA-Loaded Nanoparticles Block the Growth of Anaplastic Thyroid Cancer Xenograft. Mol. Cancer Ther..

[83] Liu C., Han Q., Liu H., Zhu C., Gui W., Yang X., Li W. (2020). Precise engineering of Gemcitabine prodrug cocktails into single polymeric nanoparticles delivery for metastatic thyroid cancer cells. Drug Deliv..

[84] Marano F., Frairia R., Rinella L., Argenziano M., Bussolati B., Grange C., Mastrocola R., Castellano I., Berta L., Cavalli R. (2017). Combining doxorubicin-nanobubbles and shockwaves for anaplastic thyroid cancer treatment: Preclinical study in a xenograft mouse model. Endocr. Relat. Cancer.

[85] Wang W., Chang J., Jia B., Liu J. (2020). The Blood Biomarkers of Thyroid Cancer. Cancer Manag. Res..

[86] Haase J., Misiak D., Bauer M., Pazaitis N., Braun J., Pötschke R., Mensch A., Bell J.L., Dralle H., Siebolts U. (2021). IGF2BP1 is the first positive marker for anaplastic thyroid carcinoma diagnosis. Mod. Pathol..

[87] Leung A., Brent G., Randolph G. (2021). Thyroid Physiology and Thyroid Function Testing. Surgery of the Thyroid and Parathyroid Glands.

[88] Giovanella L., Clark P.M., Chiovato L., Duntas L., Elisei R., Feldt-Rasmussen U., Leenhardt L., Luster M., Schalin-Jäntti C., Schott M. (2014). Thyroglobulin measurement using highly sensitive assays in patients with differentiated thyroid cancer: A clinical position paper. Eur. J. Endocrinol..

[89] Ghazy E., Kumar A., Barani M., Kaur I., Rahdar A., Behl T. (2021). Scrutinizing the therapeutic and diagnostic potential of nanotechnology in thyroid cancer: Edifying drug targeting by nano-oncotherapeutics. J. Drug Deliv. Sci. Technol..

[90] Luo L., He Y. (2020). Magnetically driven microfluidics for isolation of circulating tumor cells. Cancer Med..

[91] Xu J.Y., Handy B., Michaelis C.L., Waguespack S.G., Hu M.I., Busaidy N., Jimenez C., Cabanillas M.E., Fritsche H.A., Cote G.J. (2016). Detection and Prognostic Significance of Circulating Tumor Cells in Patients With Metastatic Thyroid Cancer. J. Clin. Endocrinol. Metab..

[92] Vanni I., Tanda E.T., Spagnolo F., Andreotti V., Bruno W., Ghiorzo P. (2020). The Current State of Molecular Testing in the BRAF-Mutated Melanoma Landscape. Front. Mol. Biosci..

[93] Milbury C.A., Zhong Q., Lin J., Williams M., Olson J., Link D.R., Hutchison B. (2014). Determining lower limits of detection of digital PCR assays for cancer-related gene mutations. Biomol. Detect. Quantif..

[94] Poole J.C., Wu S.F., Lu T.T., Vibat C.R.T., Pham A., Samuelsz E., Patel M., Chen J., Daher T., Singh V.M. (2019). Analytical validation of the Target Selector ctDNA platform featuring single copy detection sensitivity for clinically actionable EGFR, BRAF, and KRAS mutations. PLoS ONE.

[95] García-Vence M., Chantada-Vázquez M.D.P., Cameselle-Teijeiro J.M., Bravo S.B., Núñez C. (2020). A Novel Nanoproteomic Approach for the Identification of Molecular Targets Associated with Thyroid Tumors. Nanomaterials.

[96] King A.D. (2008). Imaging for staging and management of thyroid cancer. Cancer Imaging.

[97] Brauckhoff K., Biermann M. (2020). Multimodal imaging of thyroid cancer. Curr. Opin. Endocrinol. Diabetes Obes..

[98] Deng X., Shao Z., Zhao Y. (2021). Solutions to the Drawbacks of Photothermal and Photodynamic Cancer Therapy. Adv. Sci..

[99] Yang W., Liang H., Ma S., Wang D., Huang J. (2019). Gold nanoparticle based photothermal therapy: Development and application for effective cancer treatment. Sustain. Mater. Technol..

[100] Samulski T.V., Grant W.J., Oleson J.R., Leopold K.A., Dewhirst M.W., Vallario P., Blivin J. (1990). Clinical experience with a multi-element ultrasonic hyperthermia system: Analysis of treatment temperatures. Int. J. Hyperth..

[101] Wood A.K., Sehgal C.M. (2015). A review of low-intensity ultrasound for cancer therapy. Ultrasound Med. Biol..

[102] Hannah A., Luke G., Wilson K., Homan K., Emelianov S. (2014). Indocyanine green-loaded photoacoustic nanodroplets: Dual contrast nanoconstructs for enhanced photoacoustic and ultrasound imaging. ACS Nano.

[103] Liu Z., Ran H., Wang Z., Zhou S., Wang Y. (2019). Targeted and pH-facilitated theranostic of orthotopic gastric cancer via phase-transformation doxorubicin-encapsulated nanoparticles enhanced by low-intensity focused ultrasound (LIFU) with reduced side effect. Int. J. Nanomed..

[104] Wu X., Suo Y., Shi H., Liu R., Wu F., Wang T., Ma L., Liu H., Cheng Z. (2020). Deep-Tissue Photothermal Therapy Using Laser Illumination at NIR-IIa Window. Nano-Micro Lett..

[105] Xue S., Ren P., Wang P., Chen G. (2018). Short and Long-Term Potential Role of Carbon Nanoparticles in Total Thyroidectomy with Central Lymph Node Dissection. Sci. Rep..

[106] Yin C., Song B., Wang X. (2021). Identification of the Parathyroid Gland with Vasculature by Intraoperative Carbon Nanoparticles. Yangtze Med..

[107] Yan S., Zhao W., Wang B., Zhang L. (2018). Preoperative injection of carbon nanoparticles is beneficial to the patients with thyroid papillary carcinoma: From a prospective study of 102 cases. Medicine.

[108] Rao S., Wang Z., Pan C., Wang Y., Lin Z., Pan Z., Yu J. (2021). Preliminary Study on the Clinical Significance and Methods of Using Carbon Nanoparticles in Endoscopic Papillary Thyroid Cancer Surgery. Contrast Media Mol. Imaging.

[109] Pavlov A.V. (1983). Regeneration of the parathyroid glands following mechanical trauma. Arkh. Anat. Gistol. Embriol..

[110] Min Y., Wang X., Chen H., Chen J., Xiang K., Yin G. (2020). Thermal Ablation for Papillary Thyroid Microcarcinoma: How Far We Have Come?. Cancer Manag. Res..

[111] Liu Y., Ma Y., Peng X., Wang L., Li H., Cheng W., Zheng X. (2021). Cetuximab-conjugated perfluorohexane/gold nanoparticles for low intensity focused ultrasound diagnosis ablation of thyroid cancer treatment. Sci. Technol. Adv. Mater..

[112] Gao X., Li A., Zhang X., Liu P., Wang J., Cai X. (2015). Thyroid-stimulating hormone (TSH)-armed polymer–lipid nanoparticles for the targeted delivery of cisplatin in thyroid cancers: Therapeutic efficacy evaluation. RSC Adv..

[113] Paolino D., Cosco D., Gaspari M., Celano M., Wolfram J., Voce P., Puxeddu E., Filetti S., Celia C., Ferrari M. (2014). Targeting the thyroid gland with thyroid-stimulating hormone (TSH)-nanoliposomes. Biomaterials.

[114] van der Meel R., Sulheim E., Shi Y., Kiessling F., Mulder W.J.M., Lammers T. (2019). Smart cancer nanomedicine. Nat. Nanotechnol..

[115] Tran S., DeGiovanni P.J., Piel B., Rai P. (2017). Cancer nanomedicine: A review of recent success in drug delivery. Clin. Transl. Med..

[116] Yu W., Zhu L., Xu G., Song Y., Li G., Zhang N. (2016). Potential role of carbon nanoparticles in protection of parathyroid glands in patients with papillary thyroid cancer. Medicine.

[117] Shi L., Xie M., Wu G., Fan J., Guo M., Yang R., Zhang J., Zhang Y., Zhou B., Lv Z. (2020). Rapid intraoperative method for the identification of metastatic lymph nodes from thyroid carcinoma. Authorea.

